# The “ *NF-ĸ* *B interacting long noncoding RNA*” ( *NKILA*) transcript is antisense to cancer-associated gene *PMEPA1*

**DOI:** 10.12688/f1000research.6400.1

**Published:** 2015-04-22

**Authors:** Johannes M. Dijkstra, David B. Alexander

**Affiliations:** 1 Institute for Comprehensive Medical Science, Fujita Health University, Dengaku-gakubo, Toyoake, 470-1192, Japan; 2 Laboratory of Nanotoxicology Project, Nagoya City University, Tanabedohri, Nagoya, 467-8603, Japan

**Keywords:** Breast cancer, NKILA, PMEPA1, NF-ĸB, long noncoding RNA, antisense, promoter, evolution

## Abstract

This correspondence concerns a recent publication in *Cancer Cell* by Liu et al. ^1^ who analyzed a long noncoding RNA (lncRNA) that they designated “ *NKILA*”. Liu et al. found that *NKILA* (1) __is upregulated by immunostimulants, (2) has a promoter with an NF-ĸB binding motif, (3) can bind to the p65 protein of the NF-ĸB transcription factor and then interfere with phosphorylation of IĸBα, and (4) negatively affects functions that involve NF-ĸB pathways.  And, importantly, they found that (5) low *NKILA *expression in breast cancers is associated with poor patient prognosis.  However, they entirely failed to mention *PMEPA1*, a gene which runs antisense to *NKILA*, and the expression of which is associated with several tumors and which encodes a protein that participates in immune pathways.

The *PMEPA1* locus, including its promoter region, which Liu et al. ^1^ only discuss in regard to *NKILA*, is highly conserved through evolution.  Our impression is that *NKILA* emerged only later in evolution, possibly as an additional means of *PMEPA1 *regulation.  Liu et al., however, only consider direct binding between *NKILA* and NF-ĸB as the mechanism for their *in vivo* observations of *NKILA *function, but do not provide solid evidence for their model.  If *in vivo* observations by Liu et al. could be explained by *NKILA* regulation of *PMEPA1*, it would contribute to the establishment of *PMEPA1* as an important topic of cancer research.  We feel that the herein presented discussion is necessary for a correct interpretation of the Liu et al. article.

## Correspondence

**Figure 1.**
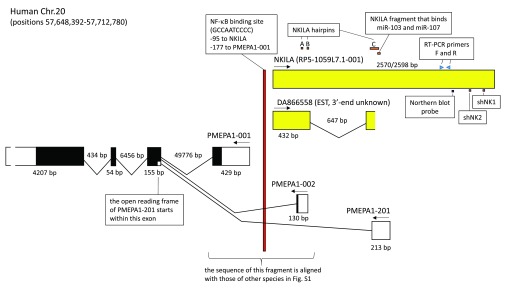
Schematic view of the *PMEPA1* plus *NKILA* region of human Chr. 20. The figure summarizes several data from the study by Liu *et al.* for *NKILA* and its promoter, while also showing overlapping transcripts that were neglected in that study. The *NKILA* transcript identified by Liu *et al.* roughly corresponds with transcript RP5-1059L7.1-001 as summarized in the GRCh38.p2 dataset of the Ensembl database ( http://www.ensembl.org/index.html). GenBank accession DA866558 (RP5-1059L7.1-002 in Ensembl) contains an expressed sequence tag (EST) which represents the 5’ end of a spliced transcript and for which the 3’ end is not known. The depicted summary of the *PMEAP1* transcripts *-001*, *-002* and *-201*, is derived from the Ensembl database and agrees with GenBank reports; for additional variations of *PMEAP1* transcripts we refer to the Ensembl database. Exons are indicated by boxes, with protein coding regions in black. The 3’ UTR of *PMEPA1* is not drawn in correct proportion to the other exon regions. Arrows indicate the direction of transcription, and genomic regions are measured in basepairs. The figure also shows from the Liu *et al.* report the positions of the NF-κB binding promoter element, the *NKILA* hairpin-prone regions, the *NKILA* region that binds miR-103 and -107, the *NKILA* binding sites for the Northern blot probe and RT-PCR primers, and the shNK1 and shNK2 regions from which sequences were derived for cloning into shRNA constructs.

Liu *et al.* ^1^ investigated breast cancer cell lines for possible association of known long noncoding transcripts with immunostimulation. They found that an unspliced lncRNA, which they designated “ *NKILA*” (represented by large yellow box in Figure 1), could be upregulated by several immune agents. However, they did not mention the existence of a reported spliced form of *NKILA* (GenBank accession DA866558, see Figure 1), or, - our major criticism - that *NKILA* is divergently transcribed from *prostate transmembrane protein, androgen induced 1* ( *PMEPA1*) and antisense to some of its transcripts (see Figure 1). One of the alternative names for *PMEPA1* is *solid tumor associated gene 1* ( *STAG1*) ^2^, and its expression was found upregulated in several tumors including breast cancer (e.g., references 2 and 3). Liu *et al.* ^1^ found that the *NKILA* promoter region contains an NF-κB binding motif ( Figure 1), which according to our analysis is rather well conserved among eutherian mammals ( Supplementary file S1). However, whereas the *PMEPA1-001* transcript open reading frame and a part of the intergenic promoter region are highly conserved through evolution, this seems to be true to a lesser degree for *NKILA* equivalent transcripts ( Supplementary file S1 and discussion therein). So, from the standpoint of evolution, a logical hypothesis for the function of the seemingly younger *NKILA* is its possible interference with *PMEPA1* expression ^4, 5^.

*PMEPA1* expression is strongly enhanced by TGF-β ^6, 7^, something which agrees with the two SMAD binding element motifs that we found conserved in its promoter ( Supplementary file S1). *PMEPA1* function is not well understood, but it is believed to encode a transmembrane protein that with its cytoplasmic domain can bind SMAD proteins and can positively affect activation of Akt ^7^. Signaling pathways involving Akt and NF-κB are known to converge ^8^, which might be relevant for a possible indirect effect of *NKILA* through *PMEPA1* on NF-κB functions. PMEPA1 levels were reported to be high in invasive MDA-MB-231 breast cancer cells, and low in non-invasive MCF-7 and T47D breast cancer cells, agreeing with observations for aggressive versus non-aggressive tumors ^9^. This is exactly opposite to the expression pattern observed by Liu *et al.* for *NKILA* in these cell lines and among tumors. *PMEPA1* knockdown has been found to be able to attenuate growth and motility of MDA-MB-231 breast cancer cells ^9^, which is interesting since Liu *et al.* ^1^ found that forced *NKILA* expression (which might knockdown *PMEPA1* expression) in MDA-MK-231 cells achieves similar effects. Consistent with these findings is the observation that high *PMEPA1* expression in breast cancer is associated with poor patient prognosis ^7^, and high *NKILA* expression with better patient prognosis ^1^. Although there are also published *PMEPA1* reports which are harder to reconcile with such a model (e.g. reference 10), and which are hard for us to validate, at least the above selected set of data suggests that *NKILA* has a negative effect on *PMEPA1* function. More research on both *NKILA* and *PMEPA1* will be necessary before conclusions can be made, but for now the possibility that *NKILA* can downregulate *PMEPA1* expression appears to be a reasonable model ^4, 5^.

Liu *et al.* ^1^ concluded that *NKILA* interferes with pathways that involve NF-κB function, and we feel that in essence this conclusion can be believably deduced from their abundant experimental data. However, mechanistically Liu *et al.* only consider a direct interaction with NF-κB components and fail to consider an indirect effect through *PMEPA1* regulation. Liu *et al.* ^1^ did find direct binding between the NF-κB component p65 and *NKILA*, but whether this can be considered as evidence of physiological specificity of *NKILA* for NF-κB is questionable. When they analyzed proteins that they could pull down with *NKILA* they only compared different NF-κB pathway components using Western blot analysis ^1^. In addition, when they quantified *NKILA* by RT-PCR on genetic material that co-precipitated with NF-κB factors, they did not exclude the possibility that they might be measuring genomic *NKILA* DNA ^1^.

Liu *et al.* ^1^ mapped the *NKILA* interaction with p65 to hairpin-prone regions A and B, and showed that the hairpin-prone region C can interact with NF-κB pathway factor IκBα (for hairpin-prone region locations see Figure 1). By mutation analysis, Liu *et al.* ^1^ found that all these three hairpin-prone regions are important for the inhibitory effect of *NKILA* on NF-κB activity. Although the relevance of this overlap is not clear, we point out that all three identified hairpin-prone regions, and also the region which Liu *et al.* ^1^ found to confer sensitivity to miRNA induced degradation, overlap with known *PMEPA1* transcript regions ( Figure 1).

As an additional remark, we would like to state that the somewhat discussable manner in the way Liu *et al.* ^1^ performed or described some of their experiments (see our comments in Supplementary file S2) does not help to convey the image of a study which is solid in its quantitative aspects. However, despite our criticism, it is only fair to state here that according to our judgement the very elaborate study by Liu *et al.* ^1^ believably shows (1) how *NKILA* can bind ( *in vitro*) to NF-κB, (2) that *NKILA* can interfere with functions that involve NF-κB pathways, and (3) that low *NKILA* expression predicts poor clinical outcome in patients with breast cancer. But they should mend the open ends, which means providing more evidence of the specificity of the *NKILA* binding to NF-κB, and to take the possible effects of *NKILA* through regulation of *PMEPA1* into consideration. In regard to the more general claim by Liu *et al.* ^1^ that there exists “a class of lncRNAs that regulate signal transduction at post-translational level”, we believe as before ^11^ that such a conclusion needs more evidence than currently has been presented. We hope that our present discussion leads to an increased interest in the *PMEPA1-NKILA* locus, because whatever mechanism may be correct, Liu *et al.* did provide evidence that this locus is clinically important in breast cancer.

## Supplementary material

*Supplementary file S1.*

Alignment of *PMEPA1-NKILA* promoter region sequences of representative animals and deduced PMEPA1 amino acid sequences for the species compared.

Click here to access the data. http://dx.doi.org/10.5256/f1000research.6400.s45984

*Supplementary file S2.*

List of issues regarding the *Liu* *et al. (2015). Cancer Cell 27, 370–381* paper which in our opinion need attention.

Click here to access the data.
