# Supplementary material for: The “ NF-ĸ B interacting long noncoding RNA” ( NKILA) transcript is antisense to cancer-associated gene PMEPA1
Source: F1000Res. 2015 Apr 22;4:96. [Version 1] doi: 10.12688/f1000research.6400.1 (PMC4431381; doi:10.12688/f1000research.6400.1)
Supplement: Peer review history

# Peer review: Referee response for version 1

**DOI:** 10.5256/f1000research.6866.r8428

**Published:** 2015-05-11

**Authors:** Pothana Saikumar

**Affiliations:** 1 Department of Pathology, University of Texas Health Science Center, San Antonio, TX, USA

Our laboratory (Saikumar) published two manuscripts on the PMEPA1/TMEPAI gene product that acts as a molecular switch in converting transforming growth factor-beta (TGF-β) from a tumor suppressor to a tumor promoter by suppressing canonical Smad signaling and promoting non-canonical PI3K/Akt signaling using breast cancer models. We have been invited to review and comment here on the correspondence paper by Dijkstra and Alexander. The correspondence titled "The NF-kB interacting long noncoding RNA (NKILA) transcript is antisense to cancer-associated gene PMEPA1” by Dijkstra and Alexander, provided an alternate explanation to the results presented by Liu *et al* in a recent publication “A cytoplasmic NF-kB interacting long noncoding RNA blocks IkB phosphorylation and suppresses the breast cancer metastasis” in Cancer Cell (27: 370-381, 2015). Although the novel finding of the later is that the long noncoding RNA designated NKILA is upregulated by immunostimulants due to the presence of a NF-kB binding motif in the promoter region of this gene which is located in the human chromosome 20q13.31. NKILA driven by NFkB acts as a negative feedback regulator of NFkB activation through a direct interaction with NF-kB p65-IkB complex, which was the basis for the negative effects on the events mediated by NF-kB signaling pathways.  Importantly, these authors either ignored or failed to notice the similarities between NKILA and PMEPA1, another gene present in the same locus of chromosome 20. A simple BLAST search would have indicated the similarities between these two genes and their locations. Interestingly, NKILA appears to fit the description of a head-to-head antisense RNA for PMEPA1.

Dijkstra and Alexander rightly noticed this omission and highlighted in their correspondence the following: i) Both NKILA and PMEPA1 genes are in anti-sense and sense orientation in the same region in chromosome 20; ii) Based on the overlap, Dijkstra and Alexander proposed a provocative hypothesis that NKILA transcript may have the potential to regulate PMEPA1.  The authors are correct in pointing out that Liu *et al.* completely overlooked the existence of PMEPA1 and did not take into account in their discussion of the mechanism involving NKILA; iii) They rightly state that indeed future experiments should verify a link between NKILA and PMEPA1. Some of the issues they raised in the supplementary file 2 were scientifically valid. This correspondence is purely theoretical and short of much needed experimental data to show that NKILA negatively regulates  PMEPA1 mRNA levels. However, in defense of the authors of the original paper, there is no evidence to suggest that immuno-stimulants like TNF-α and LPS can induce PMEPA1.

Overall the correspondence merits indexing with high importance because it will initiate research activity to identify the link between these two genes during inflammation.

I have read this submission. I believe that I have an appropriate level of expertise to confirm that it is of an acceptable scientific standard.

# Peer review: Referee response for version 1

**DOI:** 10.5256/f1000research.6866.r8429

**Published:** 2015-04-27

**Authors:** Peter G. Zaphiropoulos

**Affiliations:** 1 Department of Biosciences and Nutrition, Karolinska Institute, Huddinge, Sweden

The Dijkstra and Alexander correspondence suggests that the noncoding NKILA RNA effects on NF-κB signaling in breast cancer cells of the recent Liu *et al.* Cancer Cell publication may be partly mediated via the protein coding PMEPA1 gene, which is positioned in an antisense orientation (head to head) to the NKILA locus.

While the authors do not question the main thesis of the Liu paper, namely the direct interaction of the NKILA RNA to p65 that leads to inhibition of IκB phosphorylation, they highlight certain published data, which are indicative of a role of PMEDA1 in breast cancer and, interestingly, of a possible reciprocal regulation of PMEDA1 and NKILA.

In my opinion, the hypothesis put forward by Dijkstra and Alexander is of interest and should be experimentally addressed to test whether there is indeed an interplay between the sense-antisense PMEDA1-NKILA gene pair, which may further expand the biological roles and mechanisms of action of the NKILA noncoding RNA. In particular, I would like to see the impact of PMEDA1 depletion in the observable NKILA biological effects and vice versa the impact of NKILA depletion in PMADA1 gene expression.

Clearly, a state of validating or refuting a hypothesis by experimental means is in front of the scientific community.

I have read this submission. I believe that I have an appropriate level of expertise to confirm that it is of an acceptable scientific standard.

# Response

**Published:** 2015-04-27

**Authors:** Johannes M. Dijkstra

**Affiliations:** Fujita Health University, Japan

Dear Dr. Zaphiropoulos,

Thank you for your review and your approval. Since you are an expert in RNA functions, we are very happy that you consider our hypothesis of *NKILA *possibly affecting PMEPA1 function to be a realistic possibility.

However, you are incorrect in that we do not question the main thesis of the Liu et al. paper. We agree that for the direct *NKILA *to NF-kB binding mechanism, Liu et al. provided a lot of support by *in vitro* experiments. However, in our opinion, they did not provide the necessary conclusive *in vivo *experimental evidence for making a new model on lncRNA function. So we agree that they proved that *NKILA* can bind to NF-kB, but we say that they did not provide evidence that it does so in a specific or functional manner *in vivo*. Their paper is difficult to interpret because of the many experiments they did, combined with rather frequent incomplete descriptions of materials and methods (some of those issues we listed in our supplementary file S2). However, despite some uncertainties about what they actually did, a very critical flaw running through a large part of their paper appears to be the inability to distinguish cDNA from genomic DNA by their RT-PCR method.

We would be interested to hear from you, as an RNA expert, whether you believe that our criticism of the Liu et al. article is correct.

We are sorry that you misinterpreted our story. If the other reviewers will have a similar misinterpretation, we will have to write our criticism of the Liu et al. paper in a more explicit way.

Sincerely,

also on behalf of David B. Alexander,

Johannes M. Dijkstra

# Response

**Published:** 2015-04-29

**Authors:** Peter G. Zaphiropoulos

**Affiliations:** Karolinska Institute, Sweden

Dear Johannes (and David),

Thank you for your post.

In my reviewing of your correspondence I tried to distil its essence in terms of biological significance. In this spirit of constructive refereeing, the take-home message, in my opinion, is to test the possible interplay of the antisense protein-coding gene and the NKILA noncoding RNA, and urge you to engage in such experimental approaches to push science forward.

Concerning, your criticism of technical aspects of the Liu et al paper, I am not so sure. For example, your claim in supplementary file S2 and in your post that “RT-PCR amplification of an intronless sequence as done by the authors (see Figure 1) should consider the possibility of DNA contamination” is not in-line with the data of the Northern analysis depicted in the same Figure, panel A, which are consistent with the qRT-PCR data of Figure 1, panel C. Thus, my conclusion is that their qRT-PCR assay is robust enough and detects cDNA, as apparently their RNA preparations are essentially free of genomic DNA contamination. 

Finally, I believe that it is appropriate to contact the authors of the Liu et al paper, either directly or via Cancer Cell, to express any additional concerns that you may have.

Best wishes,

Peter

# Response

**Published:** 2015-04-29

**Authors:** Johannes M. Dijkstra

**Affiliations:** Fujita Health University, Japan

Dear Peter,

Thank you for your answer. But I like to disagree with your technical assessment of "essentially free of DNA contamination", since the methods they say to have used do not provide such material. For which of the assays, if for any, the DNA contamination had a substantial impact on the results can't be known of course, but the authors should have excluded such possibility. To my knowledge, many scientists facing similar issues are very serious to get rid of the DNA, and it is at least peculiar that in the Liu et al. study such was not tried. Awareness of the problem would also have urged them to explain the sequences of the primers for ACTB gene amplfication, since now the ACTB versus NKILA comparison could theoretically be a measure for the amount of isolated DNA. I interpret your statement of "I am not so sure" as a correct assessment of the Liu et al. paper, which in its wordings does not seem to express the normal level of scientific insecurity itself.

Personally we are currently not so interested in doing lncRNA experiments ourselves, but we will start study of PMEPA1 at the protein level. At least that may be something good that has come of it.

Best wishes,

also on behalf of David,

Johannes
